# Lack of CaBP1/Caldendrin or CaBP2 Leads to Altered Ganglion Cell Responses

**DOI:** 10.1523/ENEURO.0099-16.2016

**Published:** 2016-10-28

**Authors:** Raunak Sinha, Amy Lee, Fred Rieke, Françoise Haeseleer

**Affiliations:** 1 Department of Physiology and Biophysics, Howard Hughes Medical Institute, University of Washington, Seattle, WA 98195; 2 Departments of Molecular Physiology and Biophysics, Otolaryngology Head-Neck Surgery, and Neurology, University of Iowa, Iowa City, IA 52242; 3 Department of Physiology and Biophysics, University of Washington, Seattle, WA 98195

**Keywords:** bipolar cells, calcium-binding proteins, ganglion cells, knockout mice, light response, retina

## Abstract

Calcium-binding proteins (CaBPs) form a subfamily of calmodulin-like proteins that were cloned from the retina. CaBP4 and CaBP5 have been shown to be important for normal visual function. Although CaBP1/caldendrin and CaBP2 have been shown to modulate various targets *in vitro*, it is not known whether they contribute to the transmission of light responses through the retina. Therefore, we generated mice that lack CaBP2 or CaBP1/caldendrin (*Cabp2^–/–^* and *Cabp1^–/–^*) to test whether these CaBPs are essential for normal retinal function. By immunohistochemistry, the overall morphology of *Cabp1^–/–^* and *Cabp2^–/–^* retinas and the number of synaptic ribbons appear normal; transmission electron microscopy shows normal tethered ribbon synapses and synaptic vesicles as in wild-type retinas. However, whole-cell patch clamp recordings showed that light responses of retinal ganglion cells of *Cabp2^–/–^* and *Cabp1*^–/–^ mice differ in amplitude and kinetics from those of wild-type mice. We conclude that CaBP1/caldendrin and CaBP2 are not required for normal gross retinal and synapse morphology but are necessary for the proper transmission of light responses through the retina; like other CaBPs, CaBP1/caldendrin and CaBP2 likely act by modulating presynaptic Ca^2+^-dependent signaling mechanisms.

## Visual Abstract

**Figure:**
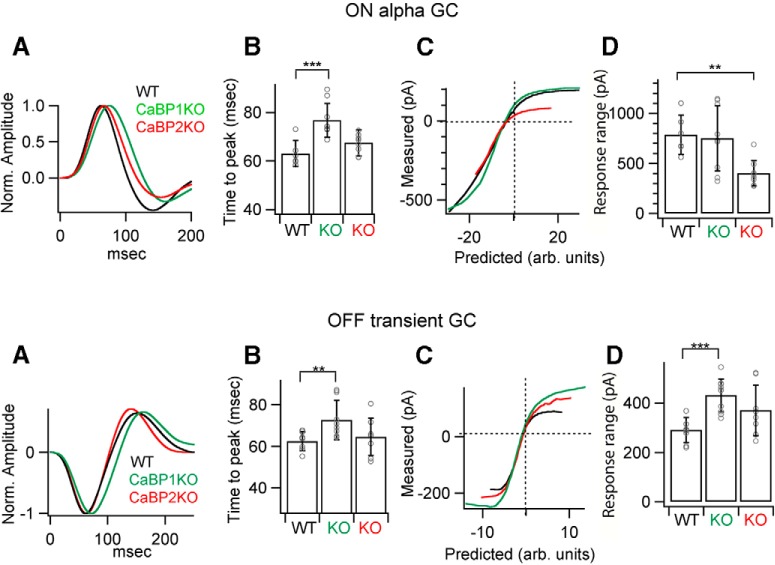

## Significance Statement

Electrical signals generated by the photoreceptors in response to incident light are processed by diverse retinal neurons before being sent to the brain. Ca^2+^ signaling controls both cellular and synaptic mechanisms that shape signals as they are transmitted through the retina. Ca^2+^-binding proteins (CaBPs), including the calmodulin-like CaBPs, exert Ca^2+^-dependent effects on specific target proteins—e.g., ion channels. To determine whether CaBP1/caldendrin and CaBP2 are important for normal retinal function, we used CaBP1/caldendrin- and CaBP2-deficient mice. Although these proteins are not required for retinal development and maintenance, CaBP1/caldendrin and CaBP2 are important for normal transfer of light signals through the retina.

## Introduction

CaBPs are neuronal Ca^2+^-binding proteins with high homology to calmodulin (CaM; Haeseleer et al., 2000). CaBPs possess four EF-hand Ca^2+^-binding domains, but unlike CaM, only three of them are capable of binding Ca^2+^. CaM and CaBPs can interact with similar targets, but their differences can underlie distinct forms of regulation. For example, CaM and CaBPs can both bind to voltage-gated Ca_v_1 L-type Ca^2+^ channels, but CaM promotes Ca^2+^-dependent inactivation, whereas CaBPs oppose this process and prolong channel opening (Zhou et al., 2004; Cui et al., 2007).

CaBPs are expressed in the retina and the cochlea; CaBP1/caldendrin is also expressed in other neuronal tissues, including the brain (Seidenbecher et al., 1998; Menger et al., 1999; Haeseleer et al., 2000, 2004; Yang et al., 2006; Cui et al., 2007; Kim et al., 2014). CaBP4 has been well characterized and is essential for normal neurotransmitter release from photoreceptors through enhanced activation of Ca_v_1.4 L-type channels (Haeseleer et al., 2004; Shaltiel et al., 2012). Mutations in the genes encoding CaBP4 and Ca_v_1.4 lead to similar visual disorders in humans and animal models (Bech-Hansen et al., 1998; Strom et al., 1998; Boycott et al., 2001; Wutz et al., 2002; Haeseleer et al., 2004; Mansergh et al., 2005; Zeitz et al., 2006; Aldahmesh et al., 2010; Khan et al., 2012; Bijveld et al., 2013; Khan, 2013). CaBP5 is expressed in rod bipolar cells and type 3 OFF and type 5 ON cone bipolar cells (Haeseleer et al., 2000; Haverkamp et al., 2003b) and is important for normal sensitivity of retinal ganglion cell light responses, likely through regulation of neurotransmitter release (Cui et al., 2007; Rieke et al., 2008; Sokal and Haeseleer, 2011).

CaBP1 and CaBP2 transcripts are alternatively spliced, resulting in short (S-) and long (L-) forms of these proteins (Haeseleer et al., 2000; Sokal et al., 2000; Haeseleer and Palczewski, 2002). A third splice variant of CaBP1, named caldendrin (CD), incorporates a different N-terminal exon and lacks the N-terminal myristoylation site present in S- and L-CaBP1 (Seidenbecher et al., 1998). For simplicity, we will use CaBP1/CD to refer to all three variants (S-CaBP1, L-CaBP1, and caldendrin) and CaBP1 to refer to S-CaBP1 and L-CaBP1. CaBP1/CD is expressed in different regions of the brain and is localized pre- and postsynaptically (Seidenbecher et al., 1998; Kim et al., 2014). In the retina, CaBP1/CD is expressed in OFF cone bipolar cells and amacrine cells (Menger et al., 1999; Haeseleer et al., 2000; Haverkamp and Wässle, 2000). CaBP1 modulates voltage-gated calcium channels (Hardie and Lee, 2016), with different effects on channel activity depending on the channel subtype (Lee et al., 2002; Zhou et al., 2004; Yang et al., 2006; Cui et al., 2007; Findeisen and Minor, 2010; Few et al., 2011). CaBP1 also regulates inositol 1,4,5-triphosphate receptors (Yang et al., 2002; Haynes et al., 2004; Li et al., 2013) and various enzymes in vitro (Haeseleer et al., 2000; Sokal et al., 2000). Caldendrin interacts with light chain 3 of microtubule-associated protein 1A, A-kinase anchoring proteins 79/150, and the Jacob protein that couples N-methyl-d-aspartate receptor signaling to the nucleus (Seidenbecher et al., 2004; Dieterich et al., 2008; Gorny et al., 2012). CaBP2 regulates voltage-gated calcium channels, inositol 1,4,5-triphosphate receptors, and various enzymes (Haeseleer et al., 2000; Sokal et al., 2000; Yang et al., 2006; Cui et al., 2007). A mutation in the *Cabp2* gene is associated with hearing impairment in humans, likely through dysregulation of the Ca_v_1.3 channels present in auditory inner hair cells, which are modulated by CaBP2 (Schrauwen et al., 2012).

Despite the identification of a large variety of interacting partners, the contributions of CaBP1/CD and CaBP2 to the transmission of light responses through the retina is unknown. In this study, we used CaBP1/CD knockout (KO; *Cabp1^–/–^*) and newly generated CaBP2 KO (*Cabp2^–/–^*) mice to investigate the importance of CaBP1/CD and CaBP2 for normal visual function. We find that both CaBP1/CD and CaBP2 regulate ganglion cell light responses and have different effects on their contrast sensitivity.

## Material and Methods

### Antibodies

Commercially available antibodies were as follows: Alexa Fluor 555 goat anti-rabbit (Invitrogen, San Diego, CA; cat. # A21429 RRID:AB_141761), Alexa Fluor 488 goat anti-rabbit (Thermo Fisher Scientific, Waltham, MA; cat. # A11034 RRID:AB_10562715), Alexa Fluor 555 goat anti-rat (Invitrogen; cat. # A21434 RRID:AB_141733), Alexa Fluor 568 goat anti-mouse IgG2a (γ2a; Innovative Research of America, Toledo, OH; cat. # A21134 RRID:AB_1500825), Alexa Fluor 488 goat anti-mouse IgG1 (γ1; Invitrogen; cat. # A21121 RRID:AB_141514), anti-calretinin (Millipore, Bedford, MA; cat. # MAB1568 RRID:AB_94259), anti-synaptotagmin 2 (Zebrafish International Resource Center, Eugene, OR; cat. # znp-1 RRID:AB_10013783), anti-Ctbp2/ribeye (BD Biosciences, Franklin Lakes, NJ; cat. # 612044 RRID:AB_399431), anti-NK3R (rabbit polyclonal, gift of Dr. Arlene Hirano, University of California–Los Angeles, Los Angeles), anti–green fluorescent protein (GFP; Abcam, Cambridge, UK; cat. # ab13970 RRID:AB_300798), anti-calsenilin (Millipore; cat. # 05-756 RRID:AB_309969), and anti-CaBP1, anti-CaBP2, and anti-CaBP5 (Haeseleer et al., 2000).

### Generation of rat anti-CaBP1/CD and rat anti-CaBP2 antibodies

Anti-CaBP1/CD and anti-CaBP2 polyclonal antibodies were raised in rats by subcutaneous immunization with purified S-CaBP1 or S-CaBP2 recombinant proteins mixed with Freund’s adjuvant (Cocalico Biologicals, Reamstown, PA). For affinity purification of rat anti-CaBP1/CD or anti-CaBP2 antibodies, purified CaBP1 or CaBP2 was coupled to cyanogen bromide–activated Sepharose (GE Healthcare, Piscataway, NJ) according to the manufacturer’s protocol. After loading of a 10-fold dilution of the sera in PBS, the columns were washed with 20 volumes of PBS. The bound antibodies were eluted with 0.1 m glycine buffer, pH 2.5, and dialyzed overnight against PBS.

### Generation and genotyping of *Cabp2* ^–/–^ mice

All animal procedures were performed in accordance with the University of Washington Animal Care and Use Committee’s regulations. A ∼2.1-kb fragment covering part of exon 2 to part of intron 5 of *Cabp2* gene was amplified first from C57Bl/6 genomic DNA by PCR with primer FH939 (5′-GTCGACTAAGTAGCTGAGACCAGAAGAGATCGAAG-3′) that was extended with a SalI restriction site and includes a stop codon in all three open reading frames and primer FH940 (5′-GGTACCAGGAGGGCTCAGTTGCTCACATTA-3′) that was extended with a KpnI site. After sequencing of this 2.1-kb fragment, it was cloned into the targeting vector SalI and KpnI opened between the neomycin phosphotransferase gene and herpes simplex virus thymidine kinase gene. The long arm of ∼5.2 kb covering the promoter region of the *Cabp2* gene upstream the ATG was amplified in two fragments. The upstream fragment of 2 kb was amplified by PCR with primer FH937 (5′-GCGGCCGCTCGTGGTTTCAGGTGCTCTACACA-3′) that was extended with a NotI site and primer FH947 (5′-TAAGGTCTTAGAGGGTCTGACAGG-3′) that covers a SpeI restriction site. A 3.2**-**kb fragment upstream of the CaBP2 initiation codon was amplified by PCR with primer FH938 (5′-ACCCAGGTTTCTGGCCTTATGTCT-3′) that also covers the SpeI restriction site and FH948 (5′-TACCGACTGACTCATGCCTAGGTT-3′) that hybridizes a few bases downstream of the CaBP2 initiation codon. All fragments were cloned in the pCRII-TOPO vector and sequenced. A tdTomato vector (originally a gift from Dr. Roger Tsien, provided by Dr. Rachel Wong) was modified by mutagenesis using QuikChange Lightning Multi Site-Directed Mutagenesis (Agilent Technologies, Santa Clara, CA) to introduce a NheI site after the SV40 polyadenylation site with primer FH1043 (5′-GTATCTTAAGGCGTAGCTAGCAAGCTTTAATATTTTGTTAAAATTCGC-3′) and delete the internal NcoI site in tdTomato with primer FH1044 (5′-CGTAATGCAGAAGAAGACGATGGGCTGGGAGGCCTCC-3′). tdTomato was then fused to the CaBP2 promoter as a fragment NcoI-NheI and transferred together in the targeting vector as NotI-BglII and BglII-NheI fragments.

**Figure 1.**
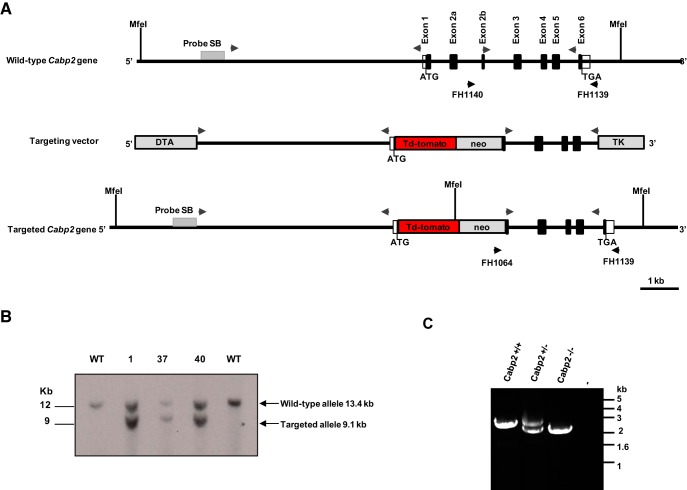
Targeting of the *Cabp2* gene. ***A***, Scheme of the mouse *Cabp2* gene with its exons. Arrows above the scheme indicate primers used to clone by PCR *Cabp2* genomic fragments. The *PGK-DTA* and *HSV-TK* cassettes were included in the targeting vector for negative selection in transfected ES cells. In the targeting vector, the *neo* cassette (positive selection) replaces exon 1 and exon 2A of the *Cabp2* gene. The targeting vector is constructed by using a ∼5-kb DNA fragment as long arm that extends upstream of the initial ATG and covers the CaBP2 promoter. tdTomato was cloned and fused on the initiation codon of CaBP2. The short arm is a ∼2.0-kb genomic fragment encompassing exon 2B to intron 5 of the *Cabp2* gene. Arrows (FH1064, FH1139, and Fh1140) below the scheme indicate primers used to select targeted *Cabp2* allele. The location of MfeI restriction site as well as the probe (probe SB) used for analysis of the targeted allele using Southern blot are also indicated. ***B***, Southern blot analysis of ES cell clones. MfeI-digested genomic DNA isolated from wild-type B6/Blu ES cells (WT) or targeted clones 1, 37, and 40 was analyzed by Southern blot with an external probe as shown in ***A*** and shows a fragment of 13.4 kb for the wild-type *Cabp2* allele and a fragment of 9.1 kb for the targeted *Cabp2* allele. ***C***, PCR-based analysis of mice for *Cabp2* targeting. A 2.3- and 2.75-kb PCR product is amplified with primers FH1140 and FH1139 as shown in ***A*** for the WT *Cabp2* allele and with primers FH1064 and FH1139 for the targeted allele, respectively.

The KpnI linearized targeting vector was electroporated into B6/BLU embryonic stem cells. Recombinant clones were selected on medium containing G418. Transfected embryonic stem (ES) cell clones were first screened through PCR analysis. To screen for homologous recombination, we used primers FH 1064 (5′-GGGTCGTTTGTTCGGATCCTCTAGAGTC-3′) located in the *neo* cassette and FH1139 (5′-TACACAGGCTCACCGAGACATCAT-3′) hybridizing approximately 163 bp downstream of the 3′ end of the short arm in the *Cabp2* gene and amplifying a fragment of ∼2.3 kb. A control PCR for the wild-type (WT) gene was made with primers FH1139 and FH1140 (5′-ACCAGGCATGGAGTTGGGTATGAA-3′) hybridizing in intron 2A, 480 bp upstream of the 5′ end of the short arm of the *Cabp2* gene and amplifying a fragment of ∼2.75 kb. Targeted disruption of the *Cabp2* gene was then confirmed by Southern blot analysis. Ten micrograms of genomic DNA was digested with MfeI and hybridized with a 0.6**-**kb 5′-end probe located 100 bp upstream of the 5′ end of the long arm (Fig. 1). This probe hybridized to a MfeI fragment of ∼13.4 kb of the WT allele or a MfeI fragment of ∼9.1 kb if the *Cabp2* gene is targeted.

One targeted ES clone was injected into C57BL/6J blastocysts. One 80% male chimera was crossed with C57BL/6J mice, and *agouti* offspring were genotyped by PCR to verify germline transmission. Confirmation of *Cabp2* gene targeting was first performed with primers FH1139, FH1140, and FH1064 as indicated above. For routine genotyping of the offspring, the WT allele was identified with primers FH1214 (5′-CCCTAAGACACCCAGACAGATGA-3′, located in intron 2A) and FH1218 (5′-GAAGTGTCAGCCAGATGGACAAA-3′, hybridizing in intron 2B) that generate a PCR product of 0.4 kb. The targeted *Cabp2* allele was identified with primers FH1218 and FH1384 (5′-TGGAGAGGCTATTCGGCTATGA-3′’, located in the *neo* cassette) that produces a PCR product of ∼1.07 kb.

### RT-PCR analysis of CaBP1 and CaBP2 transcripts

Total RNAs were isolated from mouse retina using RNeasy kit (Qiagen, Valencia, CA). Total RNA (1 μg) was subjected to first-strand cDNA synthesis using Superscript III reverse transcriptase and oligo(dT) in a volume of 20 μl according to the manufacturer’s protocol (Invitrogen). Short and long CaBP1 were amplified with common forward FH705 (5′-CACCATGGGCAACTGCGTCAAGTCG-3′) and reverse FH706 (5′-TCAGCGAGACATCATCCGGACAAAC-3′) primers. Caldendrin was amplified with primers FH1089 (5′-ACACCAATCATATCTGCCGTCTCC-3′) and FH1093 (5′-GCGATGGGGAGGAACGGGGGCT-3′). Short and long CaBP2 were amplified with common forward K122 (5′-TCCGGGCCTGGCATGGTTC-3′) and reverse FH1410 (5′-CCGAACAAATTCTTCAAAGTCAACC-3′). Amplification of glyceraldehyde 3-phosphate dehydrogenase (GAPDH) forward (5′-GAAGGGCTAATGACCACAGTCCAT-3′) and GAPDH reverse (5′-TAGCCATATTCGTTGTCGTACCAGG-3′) was used as a positive control. The PCR conditions were as follows: 94°C for 2 min, 35 cycles of 94°C for 15 s, 64°C for 30 s, and 72°C for 1 min, then 72°C for 7 min.

### Immunohistochemistry

Eyecups from 6-week-old to 3-month-old *Cabp1^–/–^*, *Cabp2^–/–^*, *Cabp1^–/–^*/*Cabp2^–/–^*, or WT mice of either sex were fixed in 4% paraformaldehyde in 0.1 m phosphate buffer, pH 7.4 (PB), for 1 h. After fixation, tissues were immersed in a graded sucrose series to 20% sucrose in 0.1 m PB and embedded in 33% optimum cutting temperature compound (Miles, Elkhart, IN) diluted with 20% sucrose in PB before being frozen. Eye tissues were cut in 12-µm sections. To block nonspecific labeling, retinal sections were incubated with 3% normal goat serum in PBST buffer (10 mm sodium phosphate, 150 mm NaCl, 0.1% Triton X-100, pH 7.4) for 20 min at room temperature. Sections were then incubated overnight at 4˚C with purified rat anti-CaBP1/CD 1:50 dilution or rat anti-CaBP2 1:20 dilution. Alexa Fluor 555–conjugated goat anti-rat IgG and Hoechst stain (1:2000) were reacted with the sections for 1 h at room temperature. The sections were rinsed in PBST and mounted with Prolong antifade reagent (Invitrogen). As indicated, some sections were incubated overnight at 4°C with a mix of rat or rabbit anti-CaBP1/CD or anti-CaBP2 antibodies and rabbit anti-NK3R at 1:500 dilution, mouse anti-Syt2 at 1:200, mouse anti-calretinin at 1:1000, chicken anti-GFP at 1:1000, mouse anti-calsenilin at 1:1000, or rabbit anti-CaBP5 at 1:200. A mix of Alexa Fluor 555–conjugated goat anti-rat IgG (1:400) and Alexa 488–conjugated goat anti-rabbit IgG (1:400), Alexa Fluor 488–conjugated goat anti-mouse IgG (1:400), or Alexa Fluor 488–conjugated goat anti-chicken IgG (1:400) was then reacted with the sections for 1 h at room temperature.

For the analysis of whole-mount retinas, the mouse retinas were fixed for 1 h in 4% paraformaldehyde in PB before dissection, incubated with 5% normal goat serum in PBST buffer overnight at 4°C, and incubated overnight at 4°C with a mix of anti-Syt2 (1:200) or anti-NK3R (1:500) and anti-Ctbp2 (1:1000). After three washes for 15 min in PBST, a mix of Alexa Fluor 568 goat anti-mouse IgG2a (γ2a) and Alexa Fluor 488 goat anti-mouse IgG1 (γ1) or a mix of Alexa Fluor 488 goat anti-mouse IgG and Alexa Fluor 555 goat anti-rabbit was reacted with the sections overnight at 4°C. After three washes in PBST, the retinal whole mounts were mounted with the photoreceptor side down in Prolong antifade reagent and analyzed under a confocal microscope (LSM710, Zeiss, Oberkochen, Germany). Immunofluorescent images were obtained with 63×/1.4, 40×/1.1, or 40×/1.3 NA objectives (Zeiss).

For quantification of the Ctbp2-labeled synaptic ribbons, confocal images of en face views in whole mounts of WT, *Cabp1^–/–^*, and *Cabp2^–/–^* retinas were acquired using a 63×/1.4 objective at a voxel size of 0.028 × 0.028 × 0.359 µm (*x-y-z*). The sublamina containing the axon terminals of cone OFF bipolar type 1 and type 2 was identified by colabeling with anti-NK3R and anti-Ctbp2. The axon terminals of cone ON bipolar type 6 were visualized by colabeling with anti-syt2 and Ctbp2. Ctbp2-labeled synaptic ribbons were counted in images of six randomly chosen 25-μm^2^ areas across the retina of four mice for each phenotype. The statistical analysis was done using the two-tailed Student’s *t*-test. Data are expressed as mean ± SE.

### Electron microscopy

Eye cups from WT, *Cabp1^–/–^*, and *Cabp2^–/–^* mice of either sex were fixed in 4% glutaraldehyde in 0.1 m sodium cacodylate buffer at 4°C. After four washes in 0.13 m PB, eyecups were dissected and postfixed in 1% buffered osmium tetroxide for 2 h on ice. After another four washes in PB, the retinas were en bloc stained in 1% uranyl acetate overnight at 4°C, dehydrated through a graded series of ethanol and two incubations in propylene oxide, and infiltrated in a 1:1 mixture of propylene oxide:epon araldite and two incubations in epon araldite, before polymerization at 60°C. Seventy-nanometer sections were cut on a Leica UC7 ultra microtome and stained with Reynolds lead citrate. The sections were viewed and images recorded on a JEOL 1230 electron microscope (JEOL, Peabody, MA). Images of bipolar cell terminals were analyzed in the outer part of the inner plexiform layer within 2–5 μm from the inner nuclear layer where type 1 and type 2 cone OFF bipolar cell axon terminals are located. Images of photoreceptor synapses with bipolar cells were taken in the outer plexiform layer.

### Electrophysiology and visual stimulation

Experiments were conducted on whole-mount retinal preparations taken from dark-adapted WT, *Cabp1^–/–^*, or *Cabp2^–/–^* mice (Schwartz et al., 2012). Isolated retina was stored in oxygenated (95% O_2_/5% CO_2_) Ames medium (Sigma-Aldrich, St. Louis, MO) at ∼32–34°C. Isolated retinas were flattened onto poly-l-lysine slides (whole mount; Schwartz et al., 2012), placed in a custom microscope, and perfused with Ames solution at a rate of ∼8 mL/min. Retinal neurons were visualized and targeted for patch-clamp recordings using infrared light (>950 nm). Voltage-clamp recordings were obtained using pipettes (2–3 MΩ) filled with an intracellular solution containing (in mm): 105 Cs methanesulfonate, 10 tetraethylammonium chloride, 20 HEPES, 10 EGTA, 2 QX-314, 5 Mg-ATP, 0.5 Tris-GTP (∼280 mOsm, pH ∼7.3 with CsOH). Alexa 594 dye (100-200 microM) was included in the intracellular solution to image the ganglion cells post recording as shown in Figs. 8 and 9. Full-field light stimuli (diameter 500 μm) were delivered to the tissue from an LED with peak spectral output at 405 nm. Recordings were performed from ventral retina. All ganglion cell recordings shown were recorded at a background light level of ∼900 R*/S cone/s, where cones dominate retinal responses. The statistical analysis was done using the two-tailed unpaired Student’s *t*-test; *p < 0.05, **p < 0.01, ***p < 0.001; error bars indicate SD.

## Results

### Generation of *Cabp2* ^–/–^ mice

Two splice variants of CaBP2, S-CaBP2, and L-CaBP2 exist and differ by the absence or presence of exon 2A (Fig. 1*A*; Seidenbecher et al., 1998; Haeseleer et al., 2000; Sokal et al., 2000). To generate *Cabp2^–/–^* mice, we designed a targeting vector to replace exon 1 and exon 2a of the *Cabp2* gene with tdTomato and a PGK-*neo* cassette as shown in Figure 1*A*. Stop codons were also introduced in all three open reading frames of exon 2B to prevent expression of any CaBP2 variants. PCR screening of ES cells identified three targeted ES cell clones for which targeting of the *Cabp2* allele was further verified using Southern blot (Fig. 1*B*). Injection of targeted ES clone 37 produced one male chimera that gave germline transmission. Targeting of the *Cabp2* gene was confirmed in the offspring by PCR on genomic DNA (Fig. 1*C*). *Cabp2^–/–^* mice were viable and did not show any apparent physiological deficits or breeding problems.

### Analysis of CaBP1/CD and CaBP2 expression in KO mice

**Figure 2.**
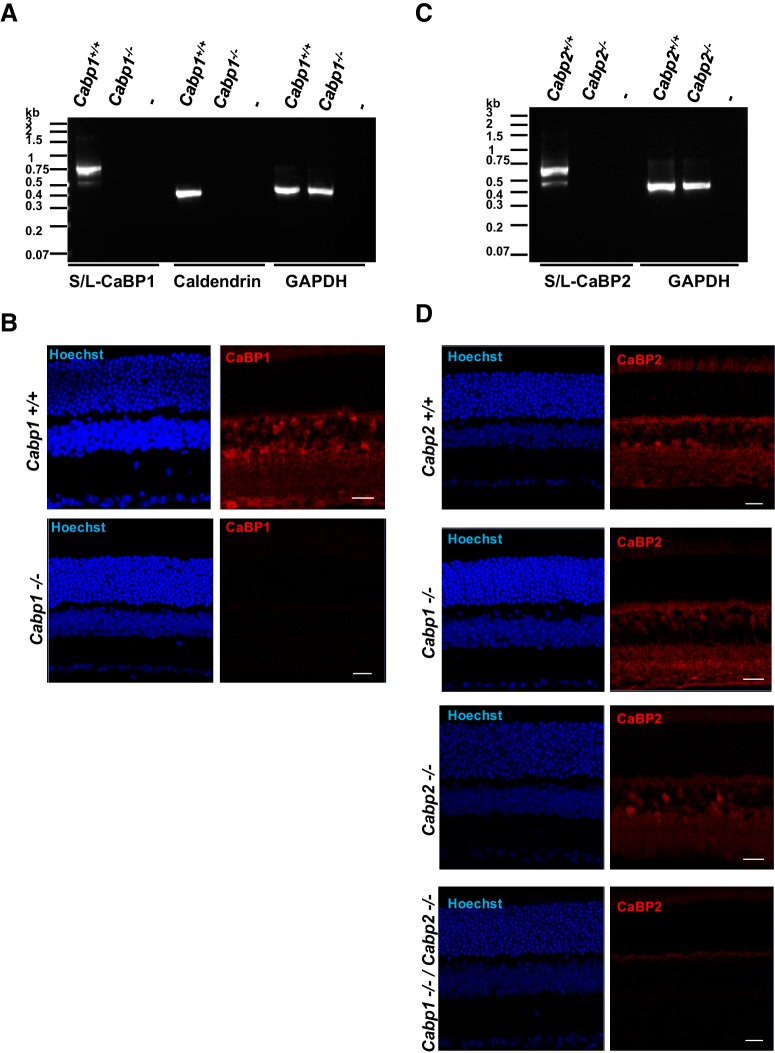
Analysis of the expression of CaBP1/CD or CaBP2 in WT, *Cabp1^–/–^*, or *Cabp2^–/–^* mice. ***A***, PCR analysis of CaBP1 and caldendrin transcripts. PCR amplification of both S-CaBP1/L-CaBP1 variants or caldendrin using RNA from *Cabp1^+/+^* or *Cabp1^–/–^* mouse retinas. PCR amplification in the absence of DNA (–) was used as a negative control, and amplification of GAPDH was the positive control. ***B***, Analysis of CaBP1/CD proteins using immunohistochemistry. Immunolocalization of CaBP1/CD in the retinas of 2-month-old *Cabp1^+/+^* and *Cabp1^–/–^* mice using rat anti-CaBP1/CD antibodies. Nuclei are labeled with Hoechst. Lack of CaBP1/CD immunoreactivity confirms targeting of the *Cabp1* gene. Scale bar: 20 μm. ***C***, PCR analysis of CaBP2 transcripts. PCR amplification of both S-CaBP2/L-CaBP2 variants from *Cabp2^+/+^* or *Cabp2^–/–^* mouse retina. Controls as described in ***A***. ***D***, Analysis of CaBP2 proteins using immunohistochemistry. Immunolocalization of CaBP2 in the retina of 2-month-old *Cabp2^+/+^*, *Cabp2^–/–^*, *Cabp1^–/–^*, and *Cabp1^–/–^*/*Cabp2^–/–^* mice using rat anti-CaBP2 antibodies. Nuclei are labeled with Hoechst. Cross-reactivity of anti-CaBP2 antibody is revealed by labeling of *Cabp2^–/–^* mouse retina with this antibody and results in staining signals in the inner retina of *Cabp2^–/–^* retina. The absence of staining in *Cabp1^–/–^*/*Cabp2^–/–^* double-KO mice demonstrates that the cross-reactivity of anti-CaBP2 antibodies is against the CaBP1/CD proteins. Specific labeling of CaBP2 in the inner retina is revealed by labeling of *Cabp1^–/–^* mouse retina with anti-CaBP2 antibodies. Scale bar: 20 μm.

The generation of *Cabp1^–/–^* mice was described by Kim et al. (2014). As a first step in the characterization of *Cabp1^–/–^* mice, we verified the absence of CaBP1/CD expression in the retina. Because exon 1 of caldendrin and exon 1 of S- and L-CaBP1 were removed in *Cabp1^–/–^* mice, all three splice variants were targeted. The absence of transcripts for all three splice variants was confirmed by reverse-transcription (RT)-PCR using retinal RNA of *Cabp1^–/–^* mice (Fig. 2*A*), whereas all three variants were observed in *Cabp1 ^+/+^* mice. As a positive control, PCR was performed with primers designed to detect GAPDH and showed transcripts in both *Cabp1 ^+/+^* and *Cabp1^–/–^* mice. The absence of CaBP1/CD proteins in the retina of *Cabp1^–/–^* mice (Fig. 2*B*) was confirmed using immunohistochemistry. Staining was not detected in *Cabp1^–/–^* mice with anti-CaBP1/CD antibodies recognizing all three splice variants.

We also verified the absence of CaBP2 expression in the newly generated *Cabp2^–/–^* mice. Using RT-PCR, transcripts were not detected for CaBP2 in *Cabp2^–/–^* retina (Fig. 2*C*) that showed transcripts for the GAPDH positive control. Using immunohistochemistry, we tested that CaBP2 proteins are not present in *Cabp2^–/–^* mice. Unfortunately, because of the high homology between CaBP1/CD and CaBP2 proteins, our anti-CaBP2 antibodies recognize both CaBP1/CD and CaBP2. As a consequence, signals were still observed in the inner nuclear layer of *Cabp2^–/–^* mouse retina immunolabeled with anti-CaBP2 (Fig. 2*D*). This residual signal was confirmed to be due to cross-reactivity of anti-CaBP2 antibodies with CaBP1/CD proteins as demonstrated by the absence of signals in *Cabp1^–/–^*/*Cabp2^–/–^* double KO retinas immunolabeled with anti-CaBP2 (Fig. 2*D*). Therefore, specific labeling of CaBP2 proteins can be visualized with our anti-CaBP2 antibodies in *Cabp1^–/–^* mouse retina. Altogether, these results confirmed that CaBP1/CD and CaBP2 are not expressed in *Cabp1^–/–^* and *Cabp2^–/–^* mice.

### Analysis of retinal morphology in *Cabp1* ^–/–^ mice

**Figure 3.**
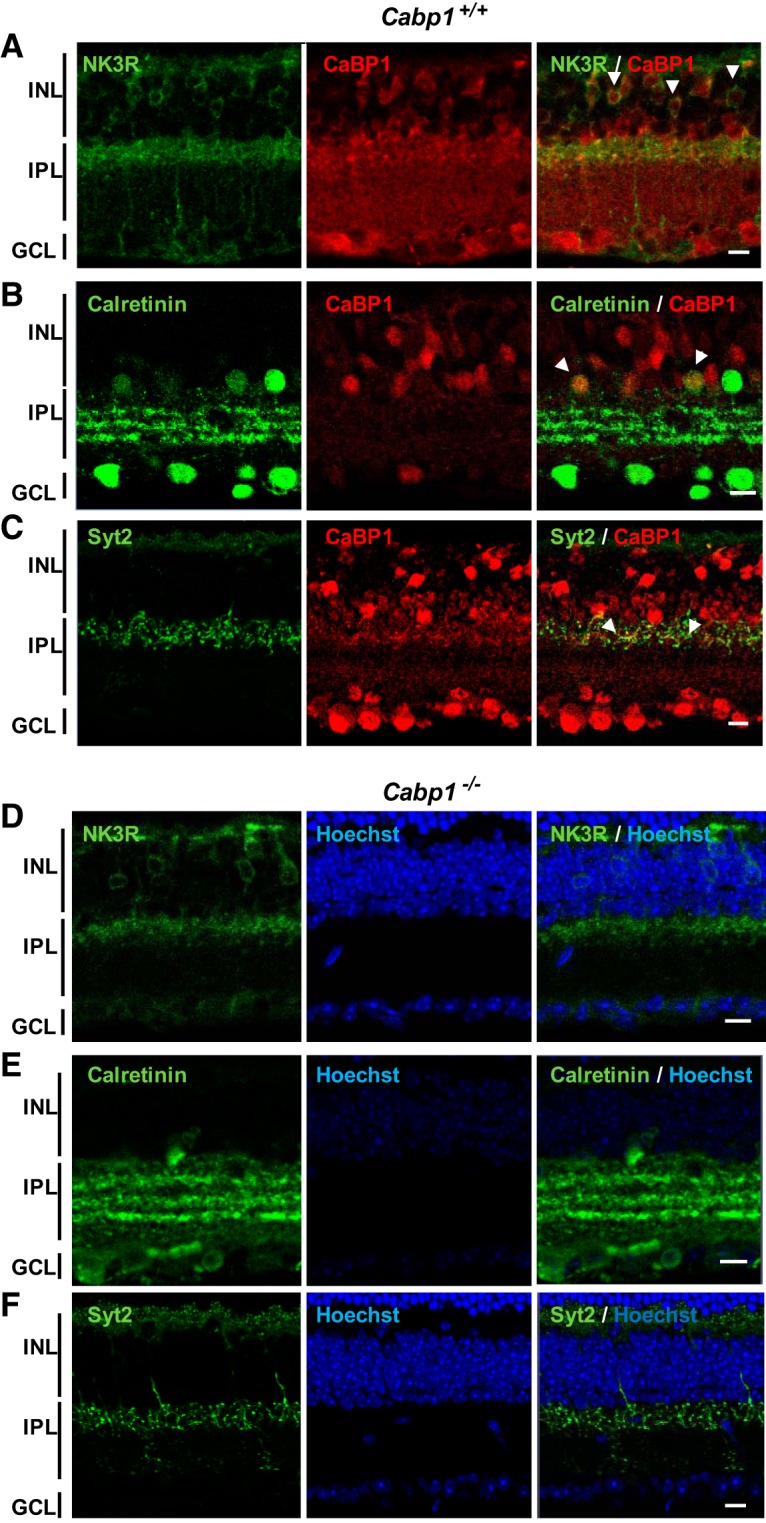
Analysis of the CaBP1/CD-expressing cells in *Cabp1^+/+^* and *Cabp1^–/–^* mice. ***A***, CaBP1/CD localizes in cells expressing NK3R in the inner retina. Analysis of *Cabp1^+/+^* mouse retina labeled with anti-NK3R (green) and rat anti-CaBP1/CD (red) using confocal microscopy. All cells labeled with anti-NK3R are also stained with anti-CaBP1/CD. Arrows in the right panels point to some of the yellow colocalization signals. INL, inner nuclear layer; GCL, ganglion cell layer. ***B***, CaBP1/CD localizes in many cells expressing calretinin in the inner retina of *Cabp1^+/+^* mouse retina. Legend as in ***A*** with anti-calretinin (green). ***C***, CaBP1/CD colocalizes with synaptotagmin 2 in the outer IPL. Legend as in ***A*** with anti-Syt2 (green). ***D–F***, Morphology of CaBP1/CD-deficient cells (NK3R stained type 1 and 2 bipolar cells, calretinin-stained amacrine cells, and Syt2-stained type 2 bipolar cells) is normal in *Cabp1^–/–^* mouse retina. Scale bars: 10 μm.

CaBP1/CD is expressed in the inner retina, specifically in amacrine cells and in OFF cone bipolar cells (Fig. 3; Table 1; Menger et al., 1999; Haeseleer et al., 2000; Haverkamp and Wässle, 2000; Haverkamp et al., 2003a). Some CaBP1/CD staining can also be observed in cells in the ganglion cell layer that may be displaced amacrine cells (Menger et al., 1999). As shown in Figure 3*A*, all cells labeled with anti–neurokinin receptor 3 (NK3R), which labels type 1 and type 2 OFF cone bipolar cells, were also labeled with anti-CaBP1/CD (Haverkamp et al., 2003a; Pignatelli and Strettoi, 2004). Synaptotagmin 2 (Syt2), another label for type 2 OFF cone bipolar cells (Wässle et al., 2009), colocalized with CaBP1/CD in the outer inner plexiform layer (IPL) (Fig. 3*C*). Some amacrine cells labeled with anti-calretinin antibodies were also immunostained with anti-CaBP1/CD (Fig. 3*B*).

**Table 1.** Identified CaBP1/CD- or CaBP2-expressing cell types in the mouse retina.

Cell type/marker	CaBP1	CaBP2
OFF type 1 BP/NK3R	+	+
OFF type 2 BP/NK3R and Syt2	+	
OFF type 3 BP (3a and 3b)/CaBP5		
OFF type 4 BP/calsenilin		
ON type 5 BP/CaBP5		
ON type 6 BP/Syt 2		+
ON type 7 BP/gustducin-GFP		
Rod BP/CaBP5		
Amacrine cells/calretinin	+	

Retinal cell types tested for the expression of CaBP1 and CaBP2 and labeled with the indicated markers.

**Figure 4.**
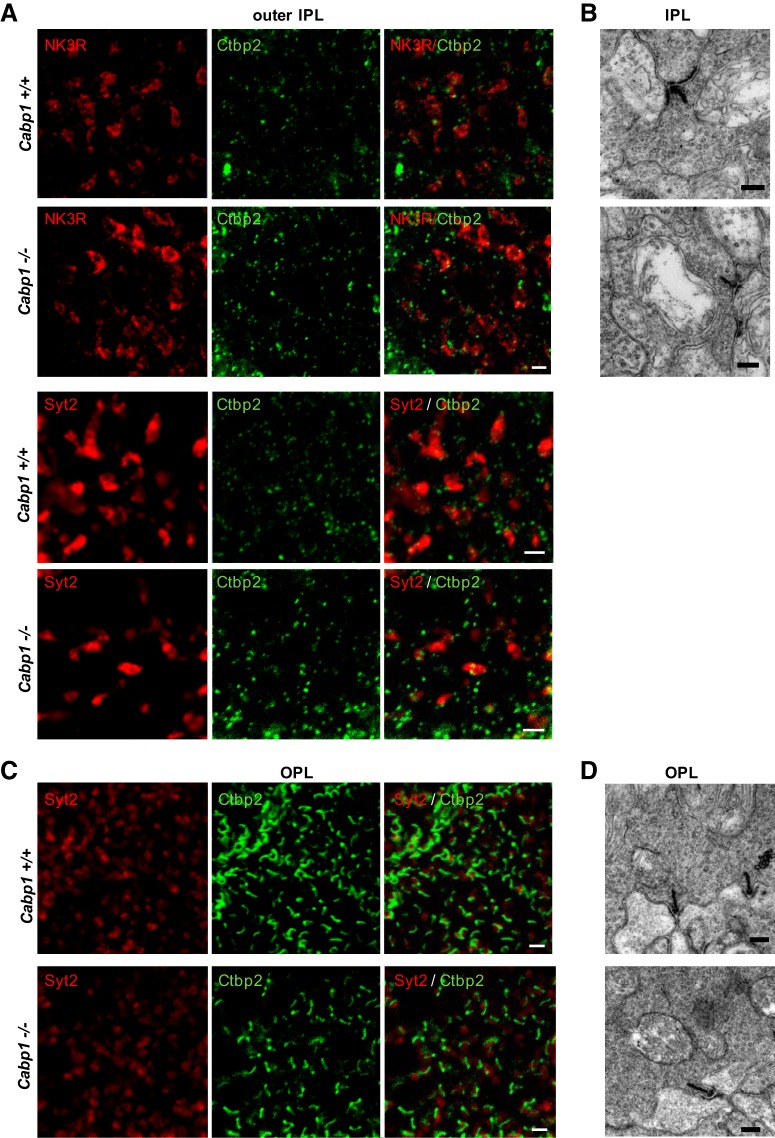
Ribbon synapse morphology in the OPL and IPL of *Cabp1^+/+^* and *Cabp1^–/–^* mice. ***A***, Confocal images showing en face views of the axon terminals of Syt2-labeled type 2 cone OFF bipolar cells (selected in IPL outer sublamina) or NK3R-labeled type 1 and 2 cone OFF bipolar cells in *Cabp1^+/+^* and *Cabp1^–/–^* retina whole mounts. Synaptic ribbons were visualized with anti-Ctbp2 (green). The axons and ribbons appear normal in both *Cabp1^+/+^* and *Cabp1^–/–^* retinas. Scale bar: 2 μm. ***B***, Representative electron micrographs of cone OFF bipolar cells in mouse retina cross-sections through the IPL within 5 μm from the inner nuclear layer. Normal ribbons and tethered vesicles are observed both in *Cabp1^+/+^* and *Cabp1^–/–^* retinas. Scale bar: 200 nm. ***C***, En face views of the dendrites of type 2 cone OFF and type 6 cone ON bipolar cells in the OPL of mouse retina whole mounts labeled with anti-Syt2 (red) and anti-Ctbp2 (green). Scale bar: 2 μm. ***D***, Representative electron micrographs of mouse retina cross-sections through the OPL. Scale bar: 200 nm.

To determine whether the absence of CaBP1/CD triggers structural changes of the retina, we analyzed the morphology of the retinal cells lacking CaBP1/CD using antibodies against these marker proteins. NK3R and Syt2 immunolabeling showed normal bipolar cell morphology and normal thickness of the INL and IPL in *Cabp1^–/–^* compared with *Cabp1 ^+/+^* retina (Fig. 3*D*, *F*). Calretinin immunostaining demonstrated normal stratification of the IPL of *Cabp1^–/–^* retina (Fig. 3*E*). To investigate whether lack of CaBP1/CD results in synaptic alterations, we analyzed retinas colabeled with anti-Syt2 or anti-NK3R and anti-ribeye/CtBP2 that labels synaptic ribbons in both the IPL and the outer plexiform layer (OPL). We analyzed the synapses in both plexiform layers because CaBP1/CD is expressed throughout the cone bipolar cells. Normal horseshoe-shaped and punctuate staining of the synaptic ribbons was observed in the IPL (Fig. 4*A*) and OPL (Fig. 4*C*) of *Cabp1^+/+^* and *Cabp1^–/–^* mouse retina. We quantified the synaptic ribbons labeled with anti-Ctbp2 in 25-μm^2^ fields of OPL and IPL of WT and *Cabp1^–/–^* mice. The density of synaptic ribbons in WT and *Cabp1^–/–^* mice did not differ significantly (Table 2).

**Table 2.** Quantification of Ctbp2-labeled ribbons.

Type	Number/25 μm^2^					
	Ribbons in OPL	*p*-value	Ribbons in NK3R-labeled outer IPL	*p*-value	Ribbons in Syt2-labeled inner IPL	*p*-value
WT	8.58 ± 0.30	1	16.33 ± 0.45	1	13.33 ± 0.42	1
*Cabp1^–/–^*	8.12 ± 0.27	0.26	16.04 ± 0.49	0.66	14.16 ± 0.53	0.22
*Cabp2^–/–^*	8.25 ± 0.21	0.36	16.75 ± 0.47	0.53	14.54 ± 0.53	0.08

Retinal whole mount, as shown in Figures 4 and 6, from four mice for each phenotype were analyzed by confocal microscopy. Ctbp2-labeled ribbons were counted in images of six areas of 25 μm^2^ for each mouse in the OPL. Ctbp2-labeled puncta were also quantified in the NK3R-labeled sublamina containing type 1 and 2 OFF bipolar cell axon terminals or Syt2-labeled sublaminae of the inner IPL containing the type 6 ON bipolar cells. Numbers represent mean ± SE (*n* = 24). Statistical comparisons with WT mice were performed using the Student’s *t*-test.

Synaptic terminals were analyzed at higher magnification using transmission electron microscopy to look for more subtle changes. Cone synapse morphology was normal, showing invagination of a pair of horizontal cells and a cone ON bipolar cell (Fig. 4*D*). Normal membrane-anchored synaptic ribbons and tethered vesicles were observed in both IPL and OPL of *Cabp1^+/+^* and *Cabp1^–/–^* retina (Fig. 4*B*, *D*). These results suggest that CaBP1/CD is not required for normal development or maintenance of overall retinal morphology.

### CaBP2 localization in bipolar cells of the retina

**Figure 5.**
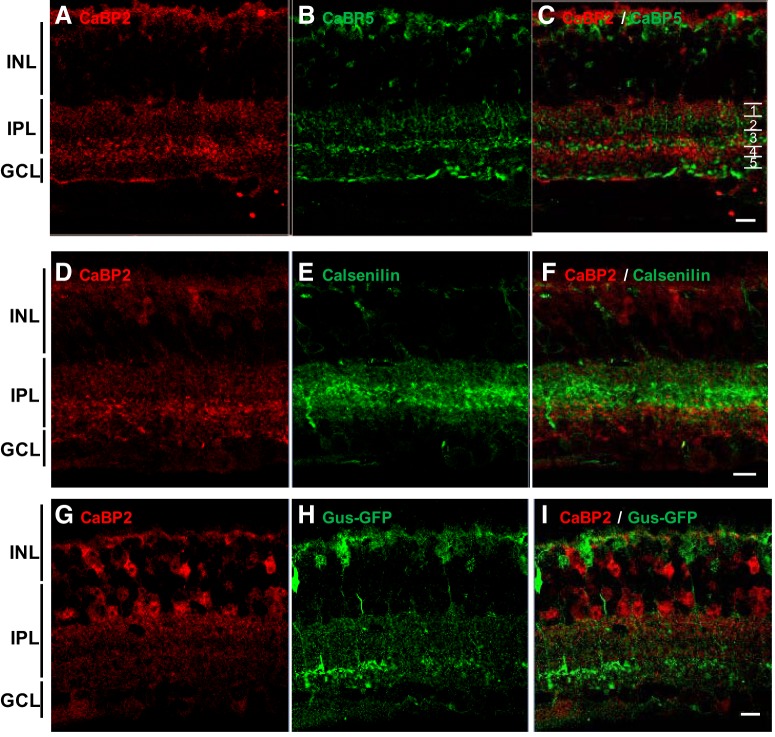
Immunolocalization of CaBP2 in mouse retina. Confocal images of *Cabp1^–/–^* (***A–F***) or Gus-GFP (***G–I***) mouse retinas double labeled for CaBP2 and CaBP5 (***A–C***), calsenilin (***D–F***), and GFP (***G–I***). Sublaminae 1–5 are indicated in ***C***. Scale bar: 10 μm.

To determine the consequences of CaBP2 deficiency, we first determined the distribution of CaBP2 in the mouse inner retina. Because our anti-CaBP2 antibody reacts with both CaBP1/CD and CaBP2 (Fig. 2*D*), and because there are no retina morphological changes in *Cabp1^–/–^*, we chose to study CaBP2 localization using *Cabp1^–/–^* mouse retina. Anti-CaBP2–labeled axons ramified in two distinct layers of the IPL, likely corresponding to ON and OFF cone bipolar cells (Fig. 2D). To determine the bipolar cell types that express CaBP2, we used antibodies against selective markers. Double-labeling experiments with antibodies against CaBP2 and CaBP5 (Fig. 5*A–C*) demonstrated that CaBP2 does not localize in bipolar cells expressing CaBP5, indicating that CaBP2 is not expressed in type 3 OFF, type 5 ON, and rod bipolar cells (Haeseleer et al., 2000; Ghosh et al., 2004). CaBP2 is also not expressed in anti-calsenilin–labeled type 4 OFF cone bipolar cells (Fig. 5*D–F*; Haverkamp et al., 2008). Gustducin-GFP mice express GFP in type 7 ON cone bipolar cells (Lin and Masland, 2005). Although our anti-CaBP2 antibody would cross-react with CaBP1/CD in gustducin-GFP retinas, none of the GFP-labeled cells in these mice were labeled with anti-CaBP2 (Fig. 5*G–I*). We can thus conclude that type 7 ON bipolar cells do not express CaBP1 and CaBP2 proteins.

**Figure 6.**
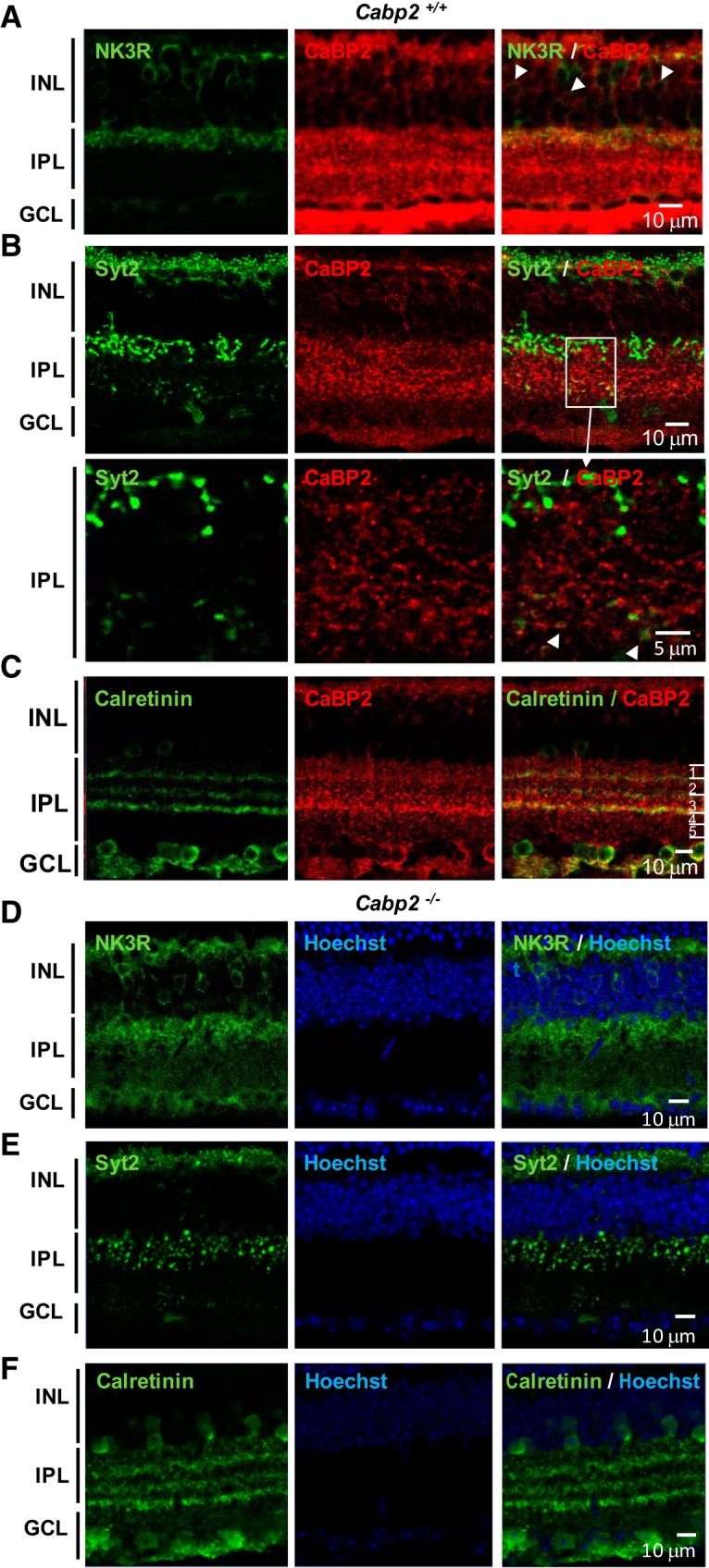
Analysis of the CaBP2-expressing cells in *Cabp2^+/+^* and *Cabp2^–/–^* mice. ***A***, CaBP2 localizes in some cells expressing NK3R in the inner retina. Analysis of *Cabp2^+/+^* mouse retina labeled with anti-NK3R (green) and rat anti-CaBP2 (red) using confocal microscopy. Some cells labeled with anti-NK3R are also stained with anti-CaBP2. Arrows in the right panels point to some of the yellow colocalization signals. INL, inner nuclear layer; GCL, ganglion cell layer. ***B***, CaBP2 localizes in some cells expressing synaptotagmin 2 in the inner retina of *Cabp2^+/+^* mice. Legend as in ***A*** with anti-Syt2 (green). The area shown at higher magnification in the bottom panels is indicated by the white box. ***C***, CaBP2-labeled cells terminate their axons in sublamina 1 and sublamina 3/4. Legend as in ***A*** with anti-calretinin (green). Sublaminae 1–5 are indicated in the right panel. ***D–F***, The overall morphology of the retina and CaBP2-deficient cells (NK3R- and synaptotagmin-labeled bipolar cells) is normal in *Cabp2^–/–^* mouse retinas.

To visualize the sublaminae of the inner plexiform layer, we labeled the mouse retina with an antibody to calretinin that displays a laminar pattern in three layers of the IPL separating sublaminae 1/2, 2/3, and 3/4 (Wässle, 2004; Fig. 6*C*) . Colabeling with anti-CaBP2 and anti-calretinin revealed that CaBP2 localizes in cells with axons in sublamina 1 and sublamina 3/4 (Fig. 6*C*). A partial overlap of the distribution of CaBP2 with NK3R indicated that either type 1 or type 2 OFF bipolar cells express CaBP2 (Fig. 6*A*). Costaining with anti-Syt2 and anti-CaBP2 antibodies showed colocalization of Syt 2 and CaBP2 in sublaminae 3/4 but not in sublamina 1 (Fig. 6*B*). This result indicates that CaBP2 is expressed in type 6 ON bipolar cells. We can also conclude from Syt 2 and NK3R labeling experiments that type 1 OFF bipolar cells express CaBP2 (Table 1). Although CaBP2 showed colocalization with Syt2 in sublamina 3/4, additional CaBP2-labeled cells appeared to have their axon arborization extending in sublamina 3/4. Because of the lack of other markers for these bipolar cells, it was not possible to identify these CaBP2-expressing cells.

### Analysis of retinal morphology in *Cabp2* ^–/–^ mice

**Figure 7.**
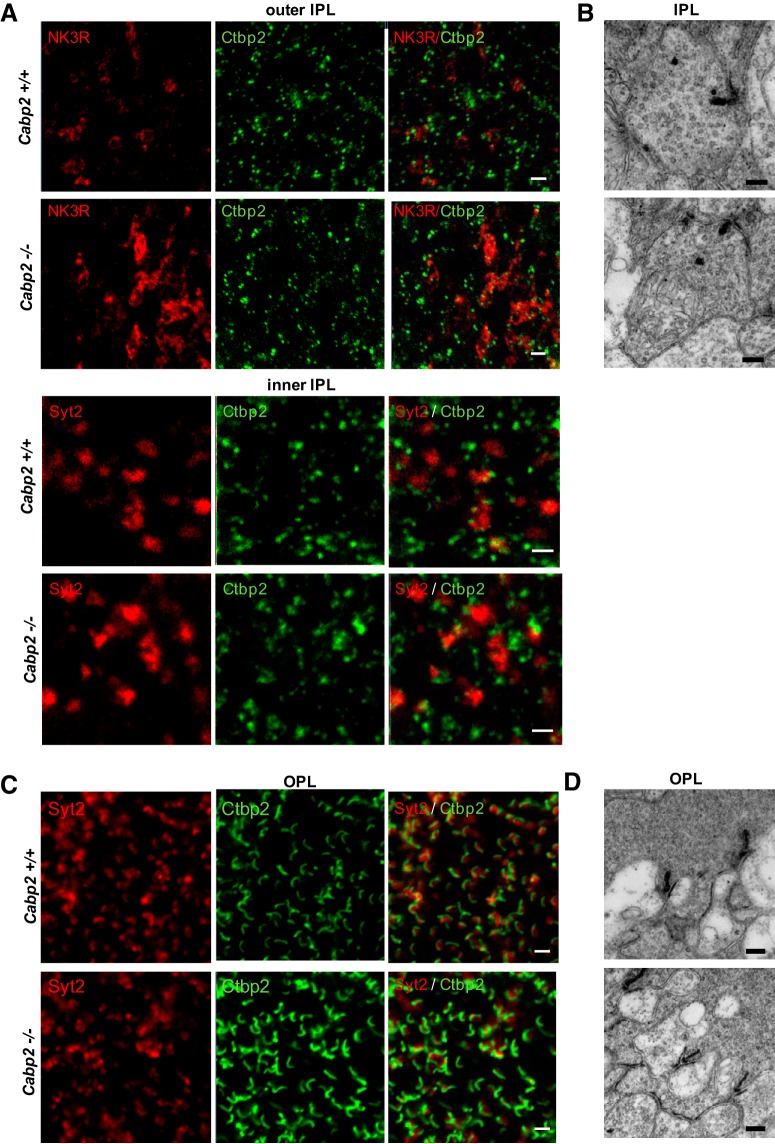
Ribbon synapse morphology in the OPL and IPL of *Cabp2^+/+^* and *Cabp2^–/–^* mice. ***A***, Confocal images of en face views of the axon terminals of Syt2-labeled type 6 cone ON bipolar cells (selected in IPL inner lamina) or NK3R-labeled cone OFF bipolar cells in *Cabp2^+/+^* and *Cabp2^–/–^* retina whole mounts. Synaptic ribbons were visualized with anti-Ctbp2 (green). The axons and ribbons of *Cabp2^+/+^* and *Cabp2^–/–^* retinas appear similar. Scale bar: 2 μm. ***B***, Representative electron micrographs of cone OFF bipolar cells in mouse retina cross-sections through the IPL within 2 μm from the inner nuclear layer. Scale bar: 200 nm. ***C***, En face views of the dendrites of type 2 cone OFF and type 6 cone ON bipolar cells in the OPL of mouse retina whole mounts labeled with anti-Syt2 (red) or anti-Ctbp2 (green). Scale bar: 2 μm. ***D***, Representative electron micrographs of cone terminals in mouse retina cross-sections through the OPL. Scale bar: 200 nm.

To determine whether CaBP2 is necessary for normal retinal development and structure, we analyzed the morphology of the CaBP2-deficient cells using antibodies against Syt2 and NK3R to look at type 6 ON and type 1 OFF bipolar cells. Overall morphology of these bipolar cells and thickness of the retinal layers did not differ noticeably between *Cabp2^+/+^* and *Cabp2^–/–^* retinas (Fig. 6). The Syt2-labeled axons of type 6 ON bipolar cells in the inner IPL and NK3R-labeled axons of OFF bipolar cells in the outer IPL showed normal morphology (Fig. 7). No difference was observed in the OPL between the Syt2-labeled dendrites of *Cabp2^+/+^* and *Cabp2^–/–^* retinas. There were also no apparent changes in the number of synapses visualized with anti-Ctbp2 in *Cabp2^–/–^* mice (Table 2). Normal synaptic ribbons and tethered vesicles were observed in both IPL and OPL of *Cabp2^+/+^* and *Cabp2^–/–^* retinas using electron microscopy (Fig. 7*B*, *D*). Cone bipolar cell and horizontal cell invaginations appeared to make a normal synapse with cone photoreceptors (Fig. 7*D*).

### Altered light responses of ganglion cells in *Cabp1* ^–/–^ and *Cabp2* ^–/–^ mice

To determine whether CaBP1/CD and CaBP2 are important for retinal function, we measured ganglion cell responses to full-field light stimuli. We selected three well-characterized mouse ganglion cell types: ON-alpha, OFF transient (OFF T), and OFF sustained (OFF S). Extensive work on these alpha-like ganglion cells has identified type-specific response properties generated from distinct circuit mechanisms (Pang et al., 2003; Murphy and Rieke, 2006; van Wyk et al., 2009; Schwartz et al., 2012). Recordings were performed at a background of 900 R*/S-cone/s, where cones dominate retinal responses.

**Figure 8.**
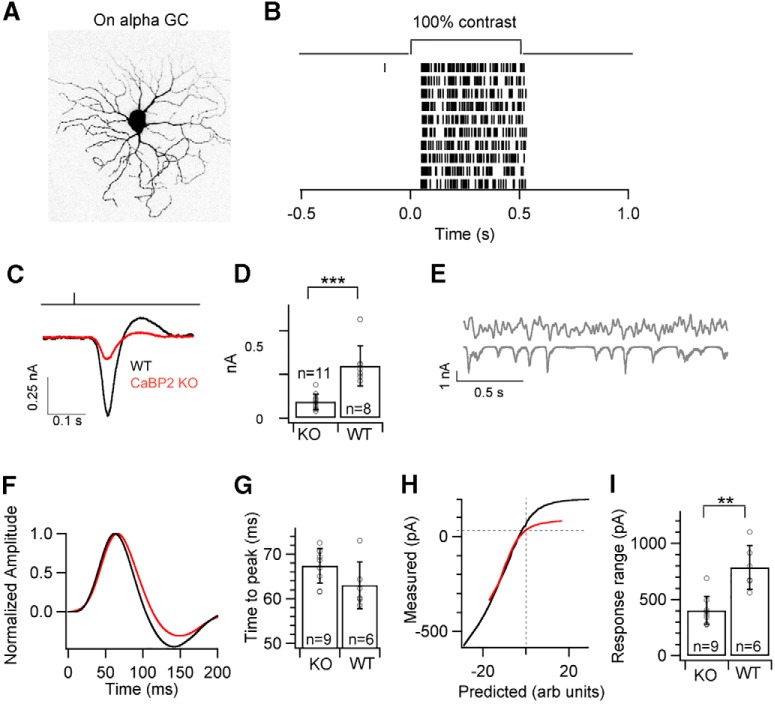
ON alpha ganglion cell responses in *Cabp2^+/+^* and *Cabp2^–/–^* mice. ***A***, Maximum-projection image of an exemplar ON alpha ganglion cell filled with dye postrecording. ***B***, Exemplar spike raster from an ON alpha ganglion cell in response to 100% contrast step. ***C***, Average excitatory synaptic currents in response to 10-ms flash of 100% contrast. ***D***, Bar graph comparing the peak amplitude of synaptic response shown in ***C*** across all cells between control and *Cabp2^–/–^.* ***E***, Linear-nonlinear (LN) model: a time-varying random stimulus and the resulting ganglion cell excitatory synaptic inputs were used to derive the linear filter and static nonlinearity that relate the stimulus to the response (top). ***F***, Average normalized linear filters for the stimuli. ***G***, Quantification of the time to peak, i.e., latency of the peaks in linear filters. ***H***, Nonlinearities of ganglion cells for the noise stimuli. ***I***, Quantification of the response range of the measured response across all cells.

Because CaBP2 is expressed in type 6 bipolar cells, and because ON alpha ganglion cells receive most excitatory synaptic input from this bipolar cell type (Morgan et al., 2011; Schwartz et al., 2012), we chose ON alphas as the primary candidate for studying perturbation in ganglion cell function in *Cabp2^–/–^* retinas (Fig. 8). Responses to a 100% contrast light flash were significantly smaller in mice lacking CaBP2 (Fig. 8*C*, *D*).

We characterized the response kinetics and contrast sensitivity using linear-nonlinear models computed from excitatory synaptic currents in response to a random noise stimulus (Chichilnisky, 2001; Kim and Rieke, 2001; Rieke, 2001). This model provides a relatively simple description of how continuous light inputs are transformed into cellular responses and provides an effective means of characterizing contrast-dependent changes in the amplitude and kinetics of the light response of a cell. The model consists of a linear filter that accounts for the time course of the response and a time-independent or “static” nonlinearity that converts the filtered stimulus into responses (excitatory synaptic currents; Fig. 8).

The linear filters of ON alpha ganglion cells were slightly slower in *Cabp2^–/–^* retinas compared with control retinas (Fig. 8*F*, *G*). The nonlinearities were strikingly different between the ON alpha ganglion cells of *Cabp2^+/+^* and *Cabp2^–/–^* retinas (Fig. 8*H*, *I*).

The response range of the responses—i.e., the difference between the maximum and minimum excitatory synaptic current evoked by the random noise stimulus—was significantly smaller for ON alpha ganglion cells in *Cabp2^–/–^* retinas compared with control cells (Fig. 8*H*, *I*).

**Figure 9.**
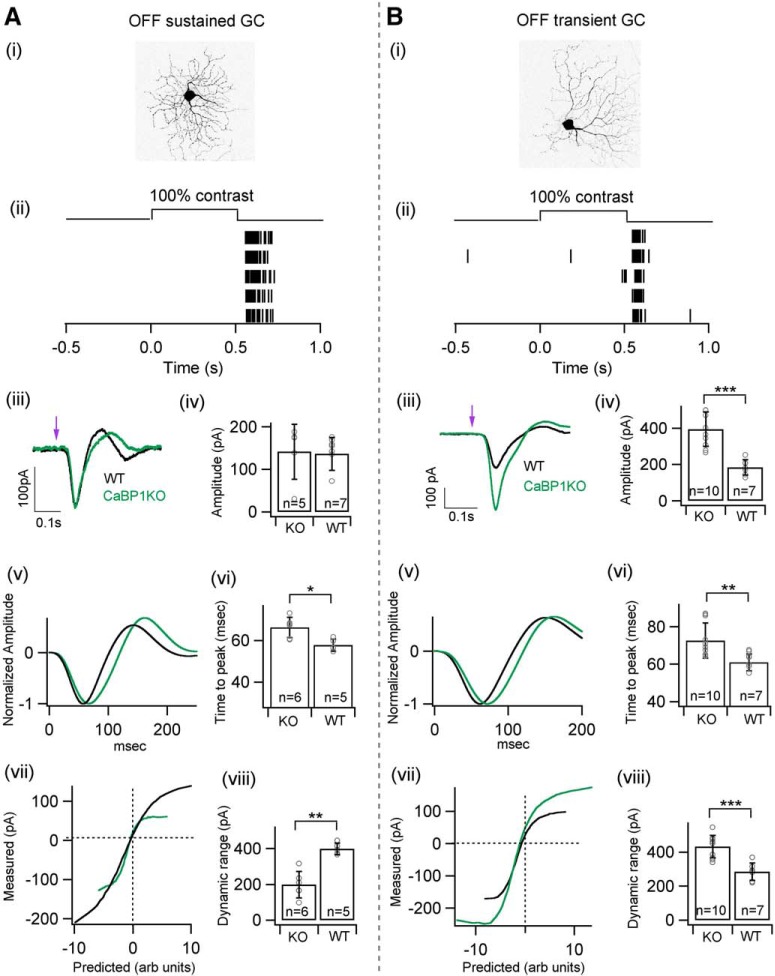
OFF alpha ganglion cell responses in *Cabp1^+/+^* and *Cabp1^–/–^* mice. ***A***, ***B (i)***, Maximum projection image of an exemplar OFF sustained and OFF transient alpha ganglion cell filled with dye postrecording. ***A***, ***B (ii)***, Exemplar spike raster in response to 100% contrast step. ***A***, ***B (iii)***, Average excitatory synaptic currents in response to a 10-ms flash of 100% contrast from control and *Cabp1^–/–^* retinas. ***A***, ***B (iv)***, Quantification of the peak amplitude of synaptic response shown in ***iii*** across all cells between control and *Cabp1^–/–^* retinas. ***A***, ***B (v)***, Linear-nonlinear (LN) model: average normalized linear filters for the stimuli. ***A***, ***B (vi)***, Quantification of the time to peak, i.e., latency of the peaks in linear filters. ***A***, ***B (vii)***, Average nonlinearities of ganglion cells for the noise stimuli. ***A***, ***B (viii)***, Quantification of the response range of the measured response across all cells between control and *Cabp1^–/–^* retinas.

**Figure 10.**
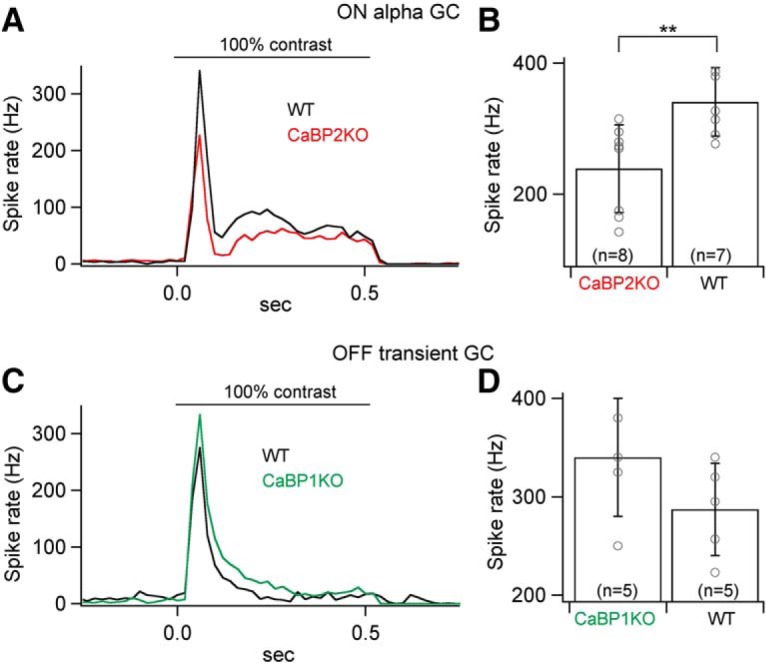
Spike responses of ON and OFF alpha ganglion cells in control, *Cabp1^–/–^*, and *Cabp2^–/–^* mice. ***A***, Average peristimulus histogram of a spike response to a 100% contrast step in ON alpha ganglion cells in *Cabp2^+/+^* and *Cabp2^–/–^* retinas. ***B***, Quantification of the peak spike rate shown in ***A***. ***C***, Average peristimulus histogram of a spike response to a 100% contrast step in OFF transient ganglion cells in *Cabp1^+/+^* and *Cabp1^–/–^* retinas. ***D***, Quantification of the peak spike rate shown in ***C***.

Because CaBP1/CD is expressed in OFF bipolar cells, we analyzed responses of OFF S and OFF T ganglion cells in *Cabp1^–/–^* retinas. Differences in lamination of OFF S and OFF T ganglion cells in the IPL suggest that they derive their excitatory inputs from different OFF bipolar cells. Responses to a light flash were larger and slower for OFF T ganglion cells compared with control retinas. Responses of OFF S ganglion cells in *Cabp1^–/–^* retinas to a light flash exhibited no significant difference in amplitude with respect to control retinas (Fig. 9*A*, *B*). The response kinetics were slower for OFF S and OFF T ganglion cells in *Cabp1^–/–^* retinas compared with controls (Fig. 9*A*, *B*). Nonlinearities were strikingly different between the OFF alpha ganglion cells from *Cabp1^–/–^* and control retinas (Fig. 9*A*, *B*). The change in the nonlinearity was opposite for OFF S and OFF T ganglion cells, with a smaller response range for *Cabp1^–/–^* OFF S ganglion cells and larger response range for *Cabp1^–/–^* OFF T ganglion cells. Changes in excitatory synaptic currents affect the spike output of the ganglion cells. Indeed, spike responses of ON alpha ganglion cell to a 100% contrast step were weaker in *Cabp2^–/–^* retinas than in control retinas (Fig. 10). Similarly, OFF T ganglion cells exhibited a stronger spike response in *Cabp1^–/–^* than in control retinas (Fig. 10).

**Figure 11.**
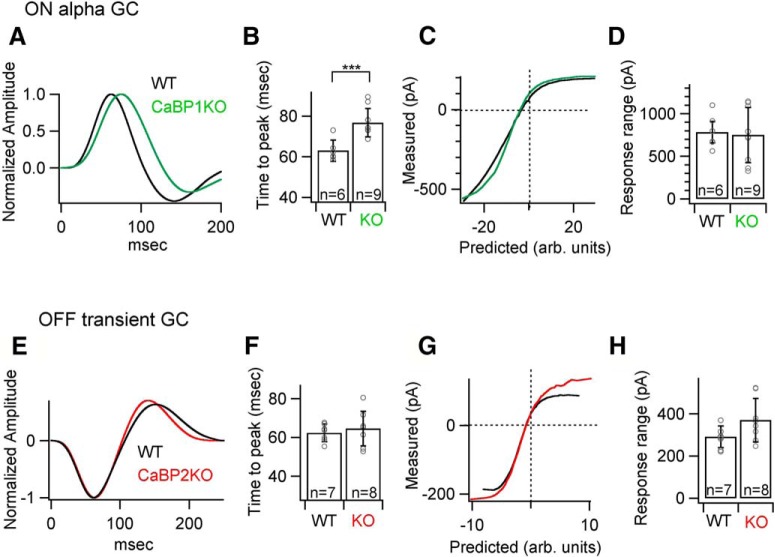
ON and OFF alpha ganglion cell responses in control, *Cabp1^–/–^*, and *Cabp2^–/–^* mice. Linear-nonlinear (LN) model: ***A***,***B***, Average normalized linear filters and quantification of the time to peak, i.e., latency of the peaks in linear filters in *Cabp1^–/–^* and control retinas of ON alpha ganglion cells. ***C***, Average nonlinearities of ON alpha ganglion cells for the noise stimuli. ***D***, Quantification of the response range of the measured response across all ON alpha ganglion cells in *Cabp1^–/–^* and control retinas. ***E***,***F***, Average normalized linear filters and quantification of the time to peak, i.e., latency of the peaks in linear filters in *Cabp2^–/–^* and control retinas of OFF transient ganglion cells. ***G***, Average nonlinearities of OFF transient ganglion cells for the noise stimuli. ***H***, Quantification of the response range of the measured response across all OFF transient ganglion cells in *Cabp2^–/–^* and control retinas.

We also compared excitatory synaptic currents of ON alpha ganglion cells in *Cabp1^–/–^* retinas and OFF T ganglion cells in *Cabp2^–/–^* retinas with corresponding control cells (Fig. 11). Responses of these cells could be affected, since CaBP1/CD is expressed in amacrine cells and CaBP2 is expressed in type 1 OFF bipolar cells. Interestingly, the response kinetics of the ON alpha ganglion cells were slower, but the response range was unchanged or slightly increased in *Cabp1^–/–^* retinas, unlike in *Cabp2^–/–^* retinas (Fig. 11). The response kinetics of the OFF T ganglion cells and the response range of responses did not change significantly in *Cabp2^–/–^* retinas (Fig. 11).

## Discussion

Ca^2+^-binding proteins modulate the cellular activities of enzymes, channels, and structural proteins in response to changes in intracellular [Ca^2+^]. Here, we determined whether the lack of CaBP1/CD or CaBP2, neuronal calcium binding proteins expressed in distinct retinal secondary neurons, results in altered transmission of light responses through the retina. We found that not only the kinetics but also the amplitude and response range of the light responses in ganglion cells are affected in the absence of CaBP1/CD or CaBP2, whereas the overall morphology of the CaBP1/CD- or CaBP2-deficient retina and synapses is not affected. These findings give a first insight into the importance of CaBP1/CD and CaBP2 for normal visual function.

### CaBP2 is expressed in cone ON and OFF bipolar cells

Our immunohistochemistry results showed that CaBP2 is expressed in type 1 cone OFF bipolar and type 6 cone ON bipolar cells. Type 1 cone ON bipolar cells are the first retinal neurons to date shown to express two different CaBPs, CaBP2, and CaBP1/CD (Figs. 3 and 6; Haverkamp et al., 2003a), since CaBPs were previously shown to be expressed in distinct neurons (Haeseleer et al., 2000, 2004; Haverkamp et al., 2003a; Ghosh et al., 2004). This raises the question of whether CaBP1/CD and CaBP2 might modulate different targets and/or have a different effect on the same target in type 1 cone OFF bipolar cells. In addition to type 1 and type 6 cone bipolar cells, other cells of the inner nuclear layers in sublamina 3/4 appeared to be immunostained with anti-CaBP2 antibody. Although we cannot exclude that type 8 and type 9 bipolar cells might express CaBP2, the axons of those cell types have been described to stratify in S4,S5 (Ghosh et al., 2004) rather than S3,S4 as observed for the CaBP2-labeled cells. Still, type 8 bipolar cells appeared to stratify broadly with the Syt2-labeled type 6 bipolar cells (Dunn and Wong, 2012). These unidentified CaBP2-labeled cells looked rather similar to the CB3,4 cells previously visualized using gene gun labeling and displaying a broad axonal arbor that extends through layers 3 and 4 (Pignatelli and Strettoi, 2004).

### Retinal morphology of *Cabp1^–/–^* and *Cabp2^–/–^* mice is normal

Immunohistochemistry analysis of *Cabp1^–/–^* and *Cabp2^–/–^* mouse retinas using specific markers showed no changes in the cell, dendritic, and axon morphologies of cells deficient for CaBP1/CD or CaBP2. Analysis of synapses by immunostaining with Ctbp2 or electron microscopy did not reveal changes in the morphology or density of synaptic ribbons or tethered synaptic vesicles. This suggests that CaBP1/CD and CaBP2 are not essential for the development and maintenance of a normal synapse or the docking of synaptic vesicles. Similar results were obtained for CaBP5 KO mice. As for *Cabp1^–/–^* and *Cabp2^–/–^* mouse retinas, no significant changes in the number of synaptic vesicles or docked synaptic vesicles was observed in *Cabp5^–/–^* mouse retina (Rieke et al., 2008). However, CaBP5 was shown to stimulate synaptic vesicle exocytosis (Sokal and Haeseleer, 2011). It is thus possible that CaBP1/CD and CaBP2 play a role in neurotransmitter release by regulating events beyond vesicle docking.

### Kinetics and amplitude of ganglion cell responses are affected in *Cabp1^–/–^* and *Cabp2^–/–^* mice

We investigated the light responses of alpha ganglion cells to determine the importance of CaBP1/CD and CaBP2 for proper transmission of light responses across the retina. Compared with responses of ganglion cells from WT retina, we observed a ∼50% increase in the amplitude of the excitatory postsynaptic currents of OFF T ganglion cells of *Cabp1^–/–^* mice and a ∼60% decrease of the excitatory synaptic currents in ON-alpha ganglion cells of *Cabp2^–/–^* mice. Because CaBP1/CD and CaBP2 are expressed throughout bipolar cells, it is possible that these altered responses originate from a defect in the processing of the light signal received from cones. Such a defect at the cone-to-cone bipolar cell synapse should affect the electroretinograms of *Cabp1^–/–^* and *Cabp2^–/–^* mice. However, the time to peak and amplitude of b-waves of electroretinograms of *Cabp1^–/–^* and *Cabp2^–/–^* mice did not differ significantly from those of WT mice (data not shown); this suggests that alterations in the transmission of the light signal likely occur downstream of the bipolar cell dendrites.

Interestingly, the two OFF alpha ganglion cell types displayed similar changes in kinetics but opposite changes in response range in *Cabp1^–/–^* mice. The opposite changes in response range could be explained if the bipolar cells that provide input to OFF S and OFF T ganglion cells express different targets for CaBP1/CD. Alternatively, CaBP1 may differently shape the synaptic input to these two types of OFF ganglions cells.

The absence of CaBP2 resulted in a decrease in the response range of the ON alpha ganglion cell response. The absence of CaBP1/CD slowed response kinetics but did not change the response range of the excitatory synaptic currents of the ON alpha ganglion cells. Because CaBP1 is not found in cone ON bipolar cells, the effect of CaBP1 deficiency on ON alpha ganglion cell excitatory inputs could result from alterations in responses of amacrine cells that provide presynaptic inhibition on type 6 bipolar cells and shape the kinetics of excitatory inputs of ON alpha ganglion cells. The complex interplay between ON and OFF pathways means that other explanations are possible as well.

Although CaBP2 deficiency results in decreased amplitude of the excitatory synaptic currents in ON alpha ganglion cells (Fig. 8), the lack of CaBP1/CD causes an increase of OFF ganglion cell responses (Fig. 9). This opposite effect on the amplitude of ganglion cell responses suggests that CaBPs may have different targets in different cell types or opposing effects on similar targets. Prominent targets of CaBPs, including CaBP1 and CaBP2, are the voltage-gated Ca^2+^ channels (Lee et al., 2002; Haeseleer et al., 2004; Zhou et al., 2004; Cui et al., 2007; Rieke et al., 2008; Schrauwen et al., 2012). Voltage-gated Ca^2+^ channels are directly involved in vesicle exocytosis and are a possible target for CaBP1/CD and CaBP2 in bipolar cells and amacrine cells (Pan et al., 2001; Habermann et al., 2003; Singer and Diamond, 2003; Balakrishnan et al., 2015). Specifically for CaBP1, it was shown that CaBP1 has opposite effects depending on the type of voltage-gated Ca^2+^ channels it modulates (Haeseleer et al., 2000; Lee et al., 2002; Zhou et al., 2004; Cui et al., 2007). Because CaBPs can both inhibit or activate voltage-gated calcium channels, it might concomitantly decrease or increase synaptic vesicle release. Using electrophysiological recordings, cone bipolar cells have been shown to express diverse Ca_v_ channels, including Ca_v_1 (L-type) and Ca_v_3 (T-type; Kaneko et al., 1989; Pan, 2000; Pan et al., 2001). T-type Ca^2+^ channels have not been shown to be modulated by calmodulin or calmodulin-like CaBPs. Although CaBP1 can interact directly with L-type and P/Q type calcium channels (Lee et al., 2002; Zhou et al., 2004; Cui et al., 2007), it could also modulate Ca^2+^ channel activity by protein kinase pathways. Indeed, in vitro experiments have shown that CaBP1 and CaBP2 can modulate calmodulin kinase II activity (Haeseleer et al., 2000) and calmodulin kinase II promotes the activity of T-type voltage-gated calcium channels (Welsby et al., 2003). The variety of voltage-gated calcium channels expressed in the inner retinal neurons and their specific regulation by CaBPs might thus account for the distinct effect of CaBP1/CD and CaBP2 deficiency on the transmission of light responses to ganglion cells.

CaBPs have also been shown to interact with other proteins playing an important role in neurotransmitter release. For example, CaBP5 interacts with Munc18-1, a component of the synaptic vesicle cycle. CaBP5 was suggested to have a role in synaptic vesicle priming by binding to a specific domain of Munc18-1 necessary for its interaction with the soluble *N*-ethylmaleimide–sensitive fusion protein attachment protein receptors (SNARE) complex. We found here that deficiency of CaBP1/CD also results in slower response kinetics than in WT animals. If CaBP1/CD were to have similar effects on synaptic vesicles priming, it could affect the kinetics of the light responses.

In conclusion, the phenotype of *Cabp1^–/–^* and *Cabp2^–/–^* mice gives us a first insight into the important role of CaBP1/CD and CaBP2 for normal transmission of the light responses through retinal circuits. Although the effects of CaBP1/CD and CaBP2 deficiency might result from a change of modulation of voltage-gated calcium channels, other cellular processes might also be altered. Future experiments investigating the interacting partners for CaBP1/CD and CaBP2 in bipolar cells will help to explain the visual phenotypes in the CaBP KO mice and the cellular functions of these proteins in the retina.

## Synthesis

The decision was a result of the Reviewing Editor Kirill Martemyanov and the peer reviewers coming together and discussing their recommendations until a consensus was reached. A fact-based synthesis statement explaining their decision and outlining what is needed to prepare a revision is listed below. The following reviewer(s) agreed to reveal their identity: Charles Ratliff

Synthesis.

Both reviewers agreed that thorough morphological characterization of localization of calcium binding proteins CaBP1 and CaBP1 together with functional and anatomical characterization of mouse knockouts lacking respective genes are significant results of potential interest to understanding retina organization. There were concerns regarding the accuracy of some claims and both reviewers agreed that lack of the quantitative analysis in the first part of the manuscript (morphology) makes the report less appealing. Particularly, a weakness of the manuscript identified by both reviewers is the uncertainty about where in the retinal circuitry CaBP1 and CaBP2 exert their influence on kinetics which limits the impact of the study. Specifically I would like to direct your attention to the following areas that would be critical for you to address should you choose to resubmit your manuscript.

1.Quantification of the morphological features examined in this study, which may be important for interpreting electrophysiological findings needs to be provided. This may include analysis of number or density of ribbons, size and number of total and docked vesicles, etc to support to support the conclusion that synaptic development is unaffected in CaBP1 KO and CaBP2 KO mice and does not contribute to the observed functional changes. The analysis needs to have the power to resolve the differences on the scale of observed functional changes.

2.It would be important to test whether changes in excitatory input to ganglion cells cause changes in the spike responses of these neurons to confirm the importance of CaBP1 and CaBP2 to the output of the retina. Ideally this would be done using the same LN analysis used for EPSCs. Alternatively, the authors could compare spike responses to a contrast step or flash among CaBP1 KO, CaBP2 KO, and wild-type mice.

3.The claims that observed deficits in GC responses originate specifically from bipolar to GC synapses in the abstract and elsewhere need to be qualified and alternative explanations need to be discussed more thoroughly. See details in a specific comment of Reviewer #2 below.

4.It would be good to include calretinin as a stratification marker in CaBP2 KO mice, as done for CaBP1 KO mice.

5.There has ben a concern regarding the low number of independent replicates in electrophysiological experiments. Given small effect sizes, it would be important to increase the n's and ensure that they represent cells from more than 3 separate animals.

In addition to these general points, the reviewers made a number of helpful suggestions, which I am providing for your reference below, in unedited form. I hope you find them helpful for improving your manuscript.

Specific Reviewer's Comments

Reviewer #1

The authors of this study explore the influence of two calcium binding proteins, CaBP1 and CaBP2, on retinal development and function. CaBP1 and CaBP2 are expressed in several classes of retinal neurons, including bipolar cells. The authors generate CaBP2 knockout (CaBP2 KO) mice and obtain CaBP1 KO mice, and verify that in these mice the respective mRNAs and proteins are removed. They then show that stratification patterns of cell-type-specific markers are unperturbed in CaBP1 KO and CaBP2 KO mice, and that ribbon synapses appear to be normal in number and ultrastructure. Finally, the authors probe the contribution of CaBP1 and CaBP2 to visual processing by recording light-evoked excitatory postsynaptic currents (EPSCs) in three large-soma ganglion cell types (ON alpha, OFF T and OFF S). Analyzing EPSCs elicited by white noise stimulation with a linear-nonlinear (LN) model they find that ganglion cell response kinetics are slowed and contrast gain is altered in CaBP1 KO and CaBP2 KO mice.

Overall this is a solid study. The quality of the data and their presentation are high, and, although some uncertainty remains about the precise site of action of CaBP1 and CaBP2, this study provides valuable new insights into the role of calcium binding proteins in neuronal signaling and the molecular mechanisms of retinal circuit function.

The following comments should be addressed:

Major:

1) The authors should quantify the density of ribbon synapses in the inner plexiform layer in their electron microscopy and / or confocal imaging (CtBP2) data, to support the important conclusion that synaptic development is unaffected in CaBP1 KO and CaBP2 KO mice and does not contribute to the observed functional changes.

2) The authors should test whether changes in excitatory input to ganglion cells cause changes in the spike responses of these neurons to confirm the importance of CaBP1 and CaBP2 to the output of the retina. Ideally this would be done using the same LN analysis used for EPSCs. Alternatively, the authors could compare spike responses to a contrast step or flash among CaBP1 KO, CaBP2 KO, and wild-type mice.

3) It would be good if the authors include calretinin as a stratification marker in CaBP2 KO mice, as they do for CaBP1 KO mice.

Minor:

1) Figure 3 top half right column should be CaBP1 rather than CaBP2

2) The results section states "We selected the three well-characterized ganglion cell types in the mouse retina..." I would suggest changing this to "We selected three well-characterized ganglion cell types in the mouse retina..."

3) I would suggest removing CaBP2 KO data from Figure 10 A-C (i.e. ON alpha) and CaBP1 KO data from Figure 10 A - C (should be D - F, i.e. OFF T). If these data were removed the authors could also include example plots of static nonlinearities, analogous to Figures 8 and 9.

Reviewer #2

The manuscript 'Lack of CaBP1 and CaBP2 leads to altered ganglion cell responses with no changes in retinal morphology' provides a novel description of the anatomical locations of Calcium binding proteins in the retina, as well as recordings from ganglion cells in wild type versus knockout mice. The localization of CaBP1 and CaBP2, especially to different subtypes of bipolar cells, will interest retinal anatomists and physiologists, and this is the most significant data in the manuscript. The ganglion cell recordings may be of interest as well, but the described effects are relatively small and difficult to interpret. Due to the strength of the anatomical work, this manuscript should be suitable for publication in a specialized retina or vision journal following substantial revision.

Major Points

1. The main research question is not well motivated.

The abstract states

"Although CaBP1/caldendrin and CaBP2 have been shown to modulate various targets in vitro, their physiological roles in vivo have not yet been investigated. Therefore, we ..."

and the introduction states

""Despite the identification of a large variety of interacting partners, the physiological roles of CaBP1 and CaBP2 in vivo remain unknown."

The research seems to be motivated by a lack of in vivo investigations into the physiological roles of CaBP1 and CaBP2, but no in vivo investigations into the physiological roles of CaBP1 or CaBP2 were performed, and there were no recordings from cells that are known to express either protein.

2. The results section is largely devoted to comparing anatomical measures in wild type versus CaBP knockout retina, but none of these comparisons are quantified.

The effects on ganglion cell receptive field kinetics are small (Fig 8G), but statistically significant, despite the low number of recordings (n=7 versus n=4 to describe a &lt;5 ms difference at p&lt;0.05 assuming the data points are Gaussian distributed; however given the overlap in values, and the apparent skew in the WT values, this Gaussian assumption should be tested). If there were a morphological effect of similar magnitude, like a 5% difference in some feature such as the number of ribbons, size of vesicles, number of docked vesicles, etc., would the authors' methods have identified this difference? This question is difficult to answer by looking at the images provided, and some effort to quantify these results is needed. Thus the conclusion in the title, that ganglion cell responses are altered but morphology is not, cannot be supported. Because the descriptions in the results section are entirely qualitative, the discussion of these results in the discussion section is largely repetitive.

3. The abstract claim that "CaBP1 and CaBP2 are important for normal transfer of light signals from bipolar cells to retinal ganglion cells" cannot be justified based on the data presented here.

There is no direct evidence that the small observed differences in ganglion cell receptive fields result from a defect at this synapse. Part of this claim is based on the idea that the ganglion cells receive majority input from a specific type of bipolar cell, and this cell type expresses a relevant CaBP. p. 16

"Since CaBP2 is expressed in Type 6 bipolar cells and ON alpha ganglion cells receive ∼70% of their synaptic input from this bipolar cell type (Schwartz et al., 2012)"

This is not correct for two reasons. First, Schwartz et al reported that 70% of identified inputs from On bipolar cells were from type 6 bipolar cells, and since it is likely that identified inputs were less than 100% of all inputs, the actual percentage is probably less than 70. Second, a significant proportion of synaptic inputs to the alpha cell are inhibitory. In a study of cat On alpha cells, 86% of input to the On alpha cell was from amacrine cells (Freed &amp; Sterling 1988). Voltage clamping a ganglion cell will not reduce the impact of these inhibitory connections to zero, or even necessarily render them negligible. These and other CaBP-expressing amacrine cells may also affect the kinetics of the bipolar cell response prior to synaptic transmission to ganglion cells, and these effects could explain the differences in ganglion cell responses independent of the function of the bipolar cell to ganglion cell synapse.

The dismissal of another possible explanation (p. 19) is not well supported.

"Because CaBP1 and CaBP2 are expressed from the dendrites to the axons of bipolar cells, it would be possible that this delayed onset of the ganglion cells responses might originate from a defect in the processing of the light signal received from cones. Such defect at the cone to cone bipolar cell synapse would likely affect the electroretinograms of Cabp1-/- and Cabp2-/- mice. However, the electroretinograms of Cabp1 -/- and Cabp2 -/- mice are normal (data not shown), suggesting that alterations in the transmission of the light signal occur downstream of the bipolar cell dendrites."

The delayed onset appears to be less than or approximately equal to 5 ms. Photoreceptor and bipolar cell signals are combined, processed and recombined by amacrine and ganglion cells, and such signal processing can magnify small kinetic differences. It is not clear that an ERG is capable of routinely detecting 5 ms differences, and any such negative result should require significant statistical power. Claiming that no difference in the ERG was observed is not sufficient to dismiss this explanation.

Another possible explanation is that CaBP expression in ganglion cells affects the ganglion cell response independently of synaptic communication. The authors have described several instances of CaBP affecting enzymes and other targets, suggesting that multiple unknown propreties of CaBP may affect multiple response properties. One possibility is that ganglion cells express CaBP, CaBP interacts structurally with potassium channels, and using Cesium or TEA in the internal recording solution to block potassium currents causes kinetic differences. There are other possible explanations as well, but if this explanation were correct, then the observed differences would fully depend on the recording solution, and a more physiological solution would produce identical receptive fields.

Minor points:

1. The manuscript needs attention to style and grammar. Paragraphs are often quite long, lack a concrete subject, and switch verb tense from sentence to sentence.

2. It is argued many times that a specific antibody does not label a specific tissue and therefore the tissue does not contain the protein targeted by the antibody. Since the literature contains many examples where this reasoning fails, such claims should be appropriately qualified, and perhaps discussed.

3. p. 3, Introduction

What is the motivation for describing similarity to calmodulin?

4. p. 4

"CaBP1 and CaBP2 transcripts are alternatively spliced, resulting in short (S-) and long (L-) forms of these proteins (Haeseleer et al., 2000). A third splice variant of CaBP1, named caldendrin, incorporates a different N-terminal exon and lacks the N-terminal myristoylation site present in the S- and L-CaBP1(Seidenbecher et al., 1998). CaBP1/CD is expressed in different regions of the brain and both caldendrin and CaBP1 are localized pre- and post-synaptically (Seidenbecher et al., 1998; Kim et al., 2014)."

The description of CaBP1 is confusing. There are three types: long form, short form, and caldendrin. Do these three types correspond to the three types discussed in the next sentence (CaBP1/CD versus CaBP1 versus caldendrin)?

5. p. 14, CaBP2 localization in bipolar cells of the retina

It would be helpful to readers if these (and similar) results were aggregated into a table with cell type as one dimension, protein as the other dimension, and a check mark or question mark if the cell type is thought to express the protein.

6. p. 16

"The calculated dynamic range of responses, a measure of the contrast gain, was significantly smaller for ON alpha ganglion cells in Cabp2-/- retinas compared to control cells (Fig. 8H-I). This suggests that the contrast gain is considerably lower for ON alpha ganglion cell excitatory synaptic responses in Cabp2-/- retinas than control."

The terms dynamic range and contrast gain must be defined. They are not the same. Neither term can be directly equated with a measured output nonlinearity from a white noise stimulus. Two ganglion cells can have the same contrast gain (if defined as the change in firing rate or membrane voltage divided by the change in stimulus contrast) and a different dynamic range (if defined as the maximum spike rate or membrane voltage minus the minimum). Similarly, two ganglion cells can have the same contrast gain and the same dynamic range, but different static nonlinearities if the signal-to-noise ratio differs between cells.

7. p. 19

"In this study, all recordings were performed on a light background where rods are saturated and thus minimally contribute to ganglion cell responses."

This statement is not universally accepted. For example, Szikra et al (2014, Roska lab) has argued that rod input affects ganglion cell responses in bright light.
